# Increased Drought Stress Tolerance in Maize Seeds by *Bacillus paralicheniformis* Halotolerant Endophytes Isolated from *Avicennia germinans*

**DOI:** 10.3390/plants15010143

**Published:** 2026-01-04

**Authors:** Dinary Eloisa Durán-Sequeda, Zamira E. Soto-Valera, Ricardo Pizarro Castañeda, María José Torres, Luz Sandys Tobias, Claudia Vergel, Alejandra Paola Quintero Linero, Hernando José Bolívar-Anillo, Ricardo Amils, Maria Auxiliadora Iglesias-Navas

**Affiliations:** 1 Grupo de Investigación Biotecnología y Genotoxicidad Ambiental (BiotecGen), Departamento de Biología, Microbiología y Afines, Facultad de Ciencias Básicas, Universidad Popular del Cesar, Diagonal 21 No. 29–56, Valledupar 200004, Colombia; 2 Centro de Investigación en Biodiversidad y Cambio Climático—ADAPTIA, Facultad de Ciencias Básicas y Biomédicas, Universidad Simón Bolívar, Diagonal 21 No. 29–56, Barranquilla 080002, Colombia; hernando.bolivar@unisimon.edu.co (H.J.B.-A.); maria.iglesias@unisimon.edu.co (M.A.I.-N.); 3 Departamento de Biología, Microbiología y Afines, Facultad de Ciencias Básicas, Universidad Popular del Cesar, Diagonal 21 No. 29–56, Valledupar 200004, Colombia; rjpizarro@unicesar.edu.co (R.P.C.); mjtorresnajera@unicesar.edu.co (M.J.T.); lstobias@unicesar.edu.co (L.S.T.); alejandraquinterol@unicesar.edu.co (A.P.Q.L.); 4 Departamento de Licenciatura en Ciencias Naturales y Educación Ambiental, Facultad de Educación, Universidad Popular del Cesar, Diagonal 21 No. 29–56, Valledupar 200004, Colombia; cvergel@unicesar.edu.co; 5 Centro de Biología Molecular Severo Ochoa (CSIC-UAM), Universidad Autónoma de Madrid, Campus Cantoblanco, 28049 Madrid, Spain; ramils@cbm.csic.es

**Keywords:** plant growth-promoting bacteria, osmotic stress adaptation, mangrove microbiome, endophyte plant interactions

## Abstract

*Avicennia germinans*, a representative of the marine coastal mangrove ecosystem, vital in the Colombian Caribbean, harbors a unique microbial diversity that could contain microorganisms with the potential to promote plant growth of agricultural species such as maize. The objective of this research was to evaluate *A*. *germinans* endophytes at different sampling sites and in diverse plant organs in order to identify the growth-promoting role of the most sodium chloride-tolerant endophyte found. These were then inoculated in maize seeds under drought stress conditions simulated by polyethylene glycol (PEG) in vitro. To this end, samples of adult *A*. *germinans* plants were collected from four mangrove ecosystems in the Colombian Caribbean. Several isolates were able to tolerate up to 15% NaCl (*w*/*v*), produce indole-3-acetic acid (IAA), show proteolytic activity, and inhibit phytopathogenic fungi. The best-performing strain, C1T-KM1901-B, was genomically identified as *Bacillus paralicheniformis* and evaluated as a bioinoculant in maize seeds under PEG-induced drought stress. Inoculation with *B*. *paralicheniformis* significantly increased germination potential and germination index of drought-resistant seeds compared to non-inoculated controls under severe drought stress conditions (40% PEG). In addition, inoculated seedlings exhibited significantly higher roots and shoot fresh and dry biomass at moderate to severe drought stress levels (15% and 20% PEG). These results are position *B. paralicheniformis* C1T-KM1901-B, isolated from *Avicennia germinans*, as a promising bioinoculant to enhance maize establishment under drought conditions.

## 1. Introduction

Plants may experience abiotic stress conditions due to several factors, which can harm the growth of crops of agricultural interest [1,2]. Drought stress is an abiotic stress that occurs when the water potential in plant tissue drops to the point that it interferes with physiological functions [3]. Low water content or a high concentration of salts in the soil solution are two factors that might produce crop drought stress. This problem, which is currently being worsened by climate change, is regarded as one of the primary global constraints on agricultural output and food security. Drought stress lowers the performance, growth characteristics, germination, seed vigor, early leaf senescence, advanced maturity, photosynthesis, chlorophyll content, and accumulation of starch in cereal crops [4,5]. Maize, one of the most significant crops in the world, was grown on approximately 203 million hectares in 2022, yielding an estimated 1.16 billion tons [6]. Numerous studies have documented maize crops’ susceptibility to drought stress, which lowers the crop’s economic output by 32% to 87%, and impacts the cereal’s vegetative, reproductive, and grain-filling stages, as well as aspects of food insecurity [7,8].

Endophytes have drawn considerable attention in recent decades due to their potential contribution to plant adaptation and enhanced abiotic stress tolerance [9]. Indeed, it has been demonstrated that using microorganisms as biological inoculants may help many different crops that experience drought stress [10,11]. Bacterial endophytes help plants manage stress by promoting the production of osmoprotectants that regulate cellular balance under challenging conditions such as salinity and drought [12]. For example, *Bacillus* sp. has been shown to reduce the activity of certain genes involved in oxidative stress, helping wheat and other similar plants cope with drought [13]. Moreover, the genus *Bacillus* is the most common type of endophyte found in halophilic plants growing in extreme environments [14]. *Bacillus* spp., isolated from agricultural soils help improve osmoregulation by increasing beneficial compounds like proline, sugars, and amino acids while reducing electrolyte leakage in maize under drought stress [15]. The *Bacillus* genus includes species that can produce hydrolytic enzymes, solubilize nutrients such as phosphorus or potassium, or produce plant growth-promoting hormones, such as auxins (e.g., indole-3-acetic acid, IAA), gibberellins, and cytokinins [16].

The Caribbean mangroves are transitional terrestrial and marine wetland ecosystems adapted to intertidal halophilic conditions, dominated by seven mangrove-forming species [17]. Several mangrove species reach optimal growth in salinities of 5 to 25% of standard seawater [18]. *Avicennia germinans* L. (Acanthaceae), also called black mangroves, is the main mangrove species of the Colombian Caribbean [19]. These plants have unique adaptations, such as pneumatophores roots [20] and halotolerant leaves [21], that allow them to survive in conditions of extreme salinity and to have a salinity-promoted propagule reproduction mechanism that is an important driver of their early emergence and growth in the mangrove [22,23]. The Colombian Caribbean has around 87,230 ha of mangrove forest [24,25]. It is estimated that more than 10% of these ecosystems has been lost in the last 30 years due to anthropogenic pressures such as road construction, water pollution, and the alteration of their hydrology, due to channeling of rivers that provide fresh water leading to a process of hyper salinization [26]. As a consequence, these pressures increase the stressful conditions these ecosystems are subject to, giving rise to hyper-salinity and accentuating drought stress conditions [27]. These new stress conditions have limited the growth of plants that previously reached more than 3 m in height, and today have been reduced to less than 1 m in height [28].

The exposure of *A*. *germinans* to natural and anthropogenic stress makes this plant a unique, yet understudied, candidate as a possible source of abiotic stress-tolerant endophytic microorganisms. The aim of this study was to characterize the plant growth promotion capacity as well as the salinity and drought tolerance of endophytic bacteria isolated from *Avicennia germinans* from the Colombian Caribbean, in order to evaluate their potential to increase the tolerance of maize to water stress conditions. To achieve this goal, a stepwise screening strategy was employed, starting with the isolation of endophytes from *A. germinans* growing under contrasting environmental conditions, followed by the selection of halotolerant strains with plant growth-promoting traits, and culminating in the genomic and functional characterization of the most promising isolate.

## 2. Results

### 2.1. Characterization of the Physicochemical Conditions of the Mangrove Environment

Table 1 presents the physicochemical characteristics of the sampling sites, including data on the plants, water, and soil from which samples of various organs of *A*. *germinans* plants were collected, including leaves, roots, and specialized structures such as pneumatophores and propagules. The water table level varied between sites, from 20 cm (KM1901) to 68 cm (SL01). Despite the low water table at KM1001, the electrical conductivity of the groundwater and the soil conductivity (41.5 mS cm^−1^) were higher at this site (146.40 mS cm^−1^) than at the rest of the sampled sites. Regarding the soil type, SL01 presents a clayey soil with 65% higher soil moisture content than the other sites, which had sandy soils and lower soil moisture. The soil pH varies slightly, being more alkaline in SL01 and CT01 (pH 8.40) and more neutral in KM1901 (pH 7.86).

### 2.2. Halotolerant Endophytic Bacteria Isolated from A. germinans

**Figure 1:**
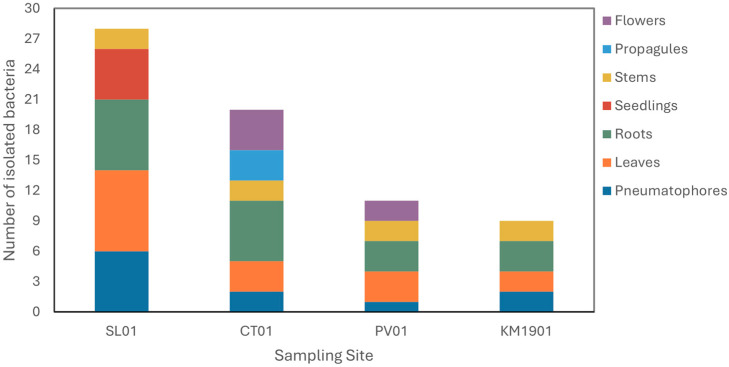
Number of endophytic bacteria isolated from tissues and organs of *A*. *germinans* in mangrove ecosystems of the Colombian Caribbean.

Sixty-eight endophytic bacteria, all tolerant to 3.5% sodium chloride, were isolated from the different organs obtained from *A*. *germinans* at four sites. Figure 1 shows the number of bacteria per organ and sampling site. Site SL01 presented the highest number of bacteria isolated from plants obtained in this study, with a total of 28 bacteria isolated. At SL01, the leaves and roots presented the highest numbers, 8 and 7, respectively, in addition to the seedlings, which were only collected at this site and from which 5 bacteria were isolated. At site CT01, all plant organs were collected. CT01 was the site with the second highest richness, with 20 isolates. At sites PV01 and KM1901, the total number of isolated bacteria was 11 and 9, respectively, with roots and leaves containing the highest number of endophytic bacteria isolated. The organs with the lowest richness were the pneumatophores, especially those from site PV01. Eight isolates were obtained from the stems, two from each sampling site.

**Figure 2:**
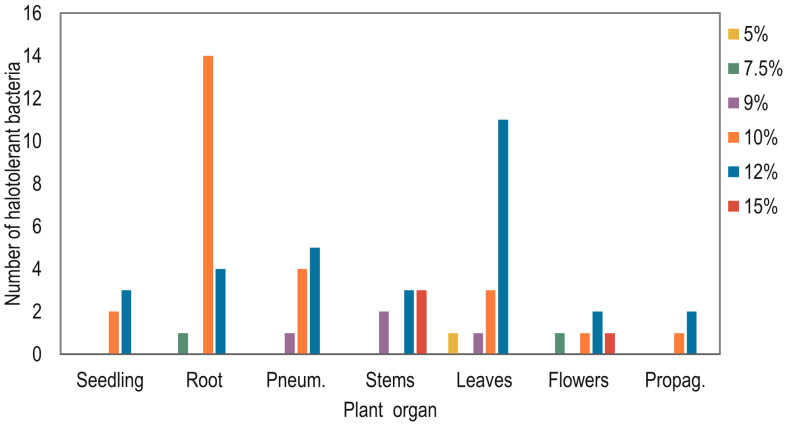
Number of halotolerant endophytic bacteria from *A*. *germinans* at maximum sodium chloride concentrations of 5%, 7.5%, 9%, 10%, 12%, and 15%, by isolation organ.

The maximum halotolerance of bacterial isolates per organ was characterized at different sodium chloride concentrations, as shown in Figure 2. All isolates were able to grow at 5% NaCl; however, Figure 2 summarizes the *maximum* NaCl concentration tolerated by each isolate. Only one isolate (from leaves) showed a maximum tolerance of 5%, whereas the remaining isolates tolerated ≥7.5% NaCl. The results show that the highest number of bacteria that tolerated a maximum sodium chloride concentration of 10% were found in the roots, and those that tolerated 12% were found in the leaves. Only in the stems and flowers were isolates found that tolerated maximum sodium chloride concentrations of 15%, with 3 isolates obtained from stems and one from flowers.

### 2.3. Characterization of Plant Growth-Promoting Activities of Halotolerant Isolates at 12 and 15% Sodium Chloride

A subset of 18 halotolerant endophytes was selected for characterization of plant growth-promoting activities (Table 2). Although a total of 34 endophytes were identified as highly halotolerant, including 30 isolates with a maximum tolerance of 12% NaCl and 4 isolates with a maximum tolerance of 15% NaCl (Figure 2), representative isolates showing the highest NaCl tolerance level for each plant organ were selected to avoid redundancy associated with similar macroscopic colony characteristics and to allow comparative analyses among tissues. As a result, 14 isolates with a maximum tolerance of 12% NaCl were included in Table 2, distributed as follows: roots (4), pneumatophores (1), leaves (6), propagules (2), and seedlings (1). In addition, 4 isolates exhibiting a maximum tolerance of 15% NaCl, obtained from stems (3) and flowers (1), were also included.

The results of this characterization show that the behavior of all these bacterial isolates compared to the tests for IAA production, proteolytic activity, phosphorus solubilization, and potassium solubilization were similar. IAA production varied between 4.0 and 19.3 μg mL^−1^. All strains showed proteolytic activity and were negative for the phosphate and potassium solubilization test. 13 of 18 isolates showed growth in nitrogen-poor media. On the other hand, the microscopic analysis of the isolates corresponded to rod-shaped bacteria, Gram-positive bacilli morphologies, all of them with the ability to form endospores.

### 2.4. Characterization of Antagonistic Activities Against Phytopathogenic Fungi of Isolates Halotolerant to 12 and 15% Sodium Chloride

**Figure 3:**
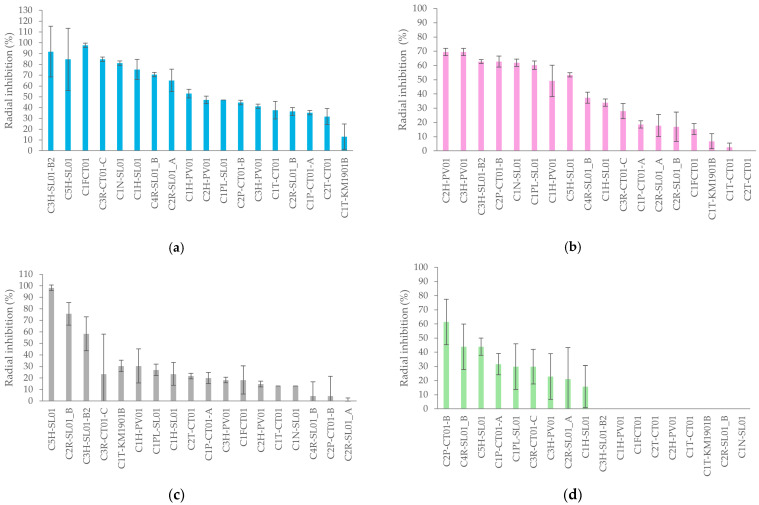
Antagonistic activity of halotolerant endophytic bacteria isolated from *A*. *germinans* against fungal phytopathogen strains *Alternaria* sp. (**a**), *Fusarium* sp. (**b**), *Botrytis cinerea* (**c**), and *Aspergillus flavus* (**d**).

The evaluation of the antagonistic activity of the *A*. *germinans* bacterial isolates are shown in Figure 3. This antagonistic activity was assessed against four strains of phytopathogenic fungi of agricultural importance: *Alternaria* sp., *Fusarium* sp., *Botrytis cinerea*, and *Aspergillus*. What was evident from this evaluation was that the interaction of each bacterial isolate assessed with each phytopathogen was different. *Alternaria* sp. was the most sensitive phytopathogen, as most isolates exhibited inhibition percentages above 40%, and several isolates (C3H-SL01-B2, C5H-SL01, and C1F-CT01) showed very high levels of radial growth inhibition (Figure 3a). In contrast, *Fusarium* sp. displayed moderate sensitivity, with eight of the eighteen strains showing inhibition values above 50%, and none achieving complete inhibition (Figure 3b). Antagonistic activity against *Botrytis cinerea* was shown by only three isolates (C5H-SL01, C2R-SL01_B, C3H-SL01-B2) none of which completely inhibited fungal growth (Figure 3c). Finally, *Aspergillus flavus* was the most resistant to the antagonism of the isolates, with only three of the isolates (C2P-CT01-B, C4R-SL01-B2, and C5H-SL01) achieving average inhibition percentages around or higher than 50%, and no cases of complete inhibition (Figure 3d).

### 2.5. Tolerance to Drought Stress by PEG of the Most Halotolerant Isolates at 12 and 15% Sodium Chloride

**Figure 4:**
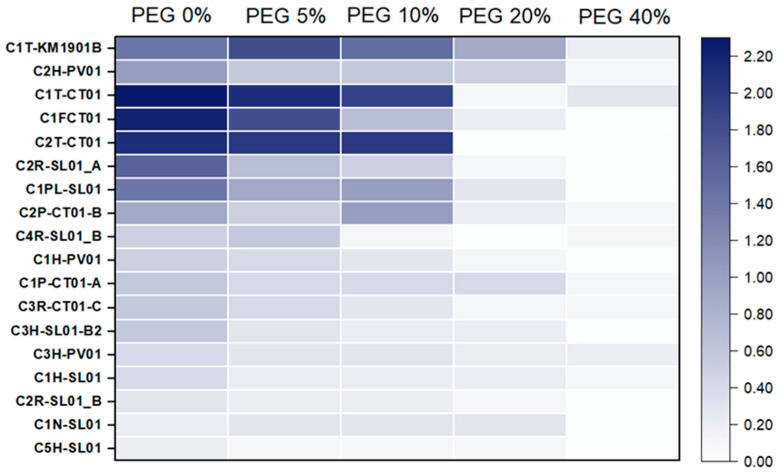
Semiquantitative assessment of drought tolerance in halotolerant endophytes isolated from *A. germinans*. Scale values indicate the mean increase in optical density (OD_600_) over time (24, 36, 48, and 72 h) relative to the initial OD of each culture.

The semiquantitative characterization of drought tolerance of halotolerant bacterial isolates obtained from *A. germinans* is shown in Figure 4. The results show that the 18 bacterial strains had a variable tolerance to the tested concentrations of PEG, which simulate stress conditions due to low water availability. However, most of the halotolerant isolates also showed tolerance to PEG at 10%. Two isolates showed consistent tolerance to drought stress up to 20% PEG, C1T-KM1901-B and C2H-PV01 strains. Only the C5HSL01 strain was sensitive to PEG and did not show growth at any of the tested concentrations. No strain was tolerant to 40% of PEG in the culture medium. 

### 2.6. Association Between Endophytic Isolated from Bacteria of A. germinans with Measured Variables

**Figure 5:**
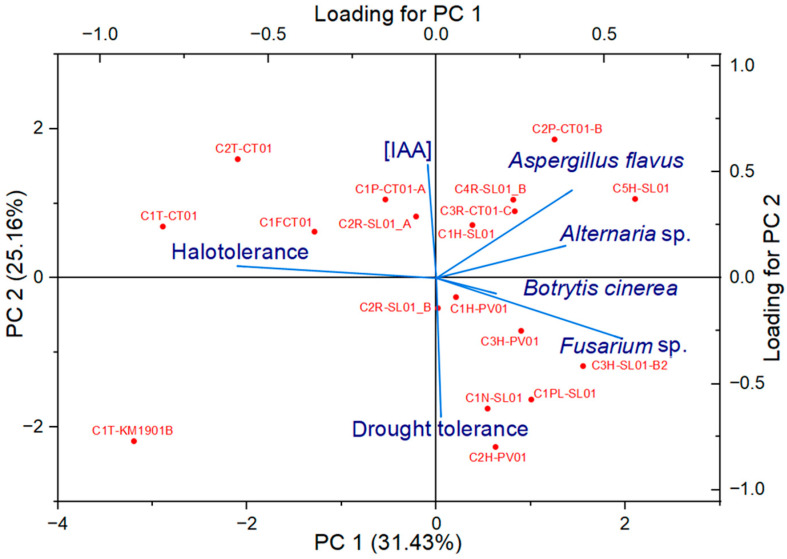
Principal Component Analysis (PCA) biplot showing the distribution of halotolerant endophytes (red dots) and the contribution of the analyzed variables (blue vectors). The bottom and left axes correspond to the PCA scores of the isolates on PC1 and PC2, while the top and right axes represent the loadings (correlation scale) of the variables.

Since the 12% and 15% sodium chloride halotolerant endophytes isolated from *A*. *germinans* showed variable IAA production behaviors, differences in antagonism against phytopathogens, and variable tolerance to drought stress by PEG, correlations between these variables were explored using a principal component analysis (PCA) which are shown in Figure 5. This analysis revealed that components 1 and 2 explained 56.6% of the observed variability. Halotolerance and antagonism against phytopathogens were the main variables that separated the strains in Component 1, while IAA production and tolerance to 20% PEG did so in Component 2. It was observed that the strains with higher halotolerance presented a lower percentage of radial inhibition against phytopathogens. On the other hand, strains with greater tolerance to drought stress by 20% PEG produced lower concentrations of IAA. The strain C1T-KM1901-B stood out, separated from the rest by its tolerance to 15% sodium chloride and 20% PEG, producing around 9 µg mL^−1^ of IAA and showing a low percentage (30%) of radial inhibition against *Alternaria* spp. This strain, isolated from the stem in one of the sites of greatest environmental and saline stress, site KM1901, was selected for its genome analysis to evaluate its potential as an endophyte to increase tolerance to drought stress in maize seeds.

### 2.7. Genomic Analysis of the Selected Strain C1T-KM1901-B Halotolerant to 15% Sodium Chloride and 20% PEG from A. germinans

To identify genes and gene products related to drought stress tolerance and plant growth promotion, the genome of isolated C1T-KM1901-B was sequenced. The genome completeness and contamination percentages were estimated to be >95% and <5%, respectively. The universal characteristics of the genome and assembly quality data are shown in Supplementary Table S1. Gene annotation resulted in a total of 4341 coding DNA sequences (CDS), 69 tRNA genes, 9 rRNA genes, and 1 tmRNA gene. The number of genes with assigned functions after annotation was 2440, corresponding to a percentage of 56.2%.

Based on the average nucleotide identity (ANI) and the average amino acid identity (AAI), the genome of the C1T-KM1901-B strain corresponds to the species *Bacillus paralicheniformis* with a 97.49% and 98% identity, respectively.

According to the annotation by Prokka, the isolate *B*. *paralicheniformis* C1T-KM1901-B presented protein sequences for abiotic stress tolerance and plant growth promotion (Table 3) supported by its identity (>35%), query coverage (>50%), and E value (<10^−5^). Supplementary Table S2 of the Supplementary Materials details all proteins included in the search and those considered absent. Among the protein sequences that stand out in Table 3 are those related to osmotic regulation (*mtnA*, *speE*), production of osmoprotectants such as proline and glycine betaine (*ectB*, *gbsB*, *codA*), proteins sequences involved in the detoxification of reactive oxygen species (*tpx*, *kat*E, *sod*A), and the biosynthesis of phytohormones such as indole-3-acetic acid (*iaaM*). In addition, proteins involved in the solubilization of phosphates and potassium (*phoA*, *gnt*P) and the synthesis of siderophores (*ent*C, *dhb*C), essential for nutrient uptake in poor soils, were identified.

**Figure 6:**
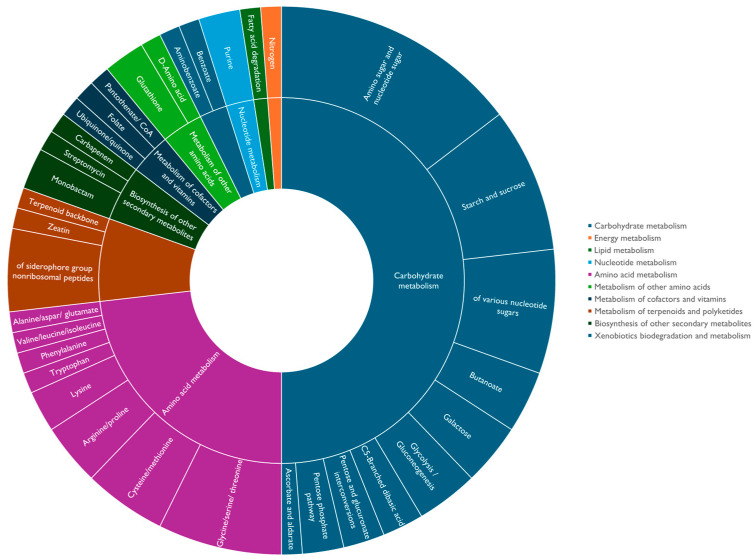
Pathway of KEGG Orthology of *B. paralicheniformis* C1T-KM1901-B protein sequences shown in Table 3.

Table 3 presents the gene annotation of the *B*. *paralicheniformis* C1T-KM1901-B genome, obtained from the KEGG (Kyoto Encyclopedia of Genes and Genomes) database. This allowed the identification of the KO terms (KEGG Orthology) and their associated molecular functions. Figure 6, on the other hand, shows the functional orthologs associated with these terms, highlighting the crucial role of carbohydrate metabolism in the processes of drought stress tolerance. The results of this functional annotation of KO genes mainly highlight the importance of carbohydrate metabolism in the mechanisms of tolerance to drought stress. It was found that 41 of the evaluated protein sequences participated in the metabolism of amino sugars, starch, galactose, and pentoses, among others; 22 in the metabolism of amino acids such as glycine, cysteine, arginine, proline, and tryptophan, among others; 6 in the metabolism of terpenoids and polyketides such as zetain and siderophores; among other sequences associated with other types of metabolism.

### 2.8. Evaluation of the Effect of Bacillus paralicheniformis C1T-KM1901-B on Maize Seed Germination Under Drought Stress

Evaluating the potential of endophytic bacteria, such as *B. paralicheniformis* C1T-KM1901-B, to enhance seed tolerance to drought conditions could benefit sustainable agriculture today. Table 4 compares germination rates and other indicators related to drought resistance under different concentrations of PEG, both in seeds treated with water and in seeds treated with the endophytic bacteria *B. paralicheniformis* C1T-KM1901-B. For germination rate and germination potential, it was observed that seeds treated with the bacteria showed higher percentages at PEG concentrations from 0% to 15%, compared to untreated seeds. However, these percentages decrease significantly with 20% and 40% PEG concentrations. Germination potential showed statistically significant (*p*-value < 0.05) differences in seeds treated with *B. paralicheniformis* under PEG at 40%, compared to untreated seeds. While seeds with the endophyte showed some germination capacity at extremely high concentrations of PEG (40%), seeds treated with water do not germinate. However, the germination rate did not show statistically significant differences under these conditions.

Regarding drought resistance and vigor indices, the results were similar. The treatment with the bacteria produced a higher germination rate at low and moderate PEG concentrations, but this was drastically reduced at higher concentrations. Finally, the results of the root-shoot ratio indicate that at low and moderate PEG concentrations, this ratio is higher in seeds treated with *B. paralicheniformis* C1T-KM1901-B. Nevertheless, at extremely high PEG concentrations, the ratio was considerably reduced. These results strongly suggest that treatment with the C1T-KM1901-B endophyte bacteria from *A. germinans* improves germination and seed yield under drought stress conditions up to 20% PEG. Higher PEG concentrations negatively affect seed and bacterial growth.

**Figure 7:**
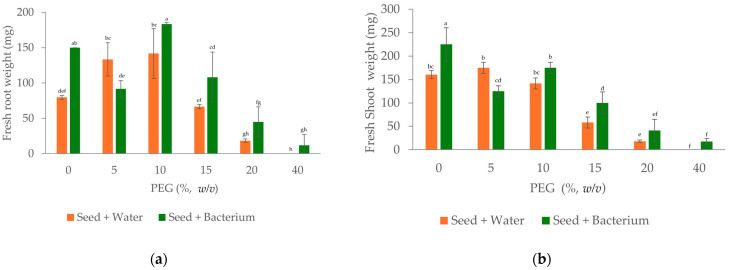

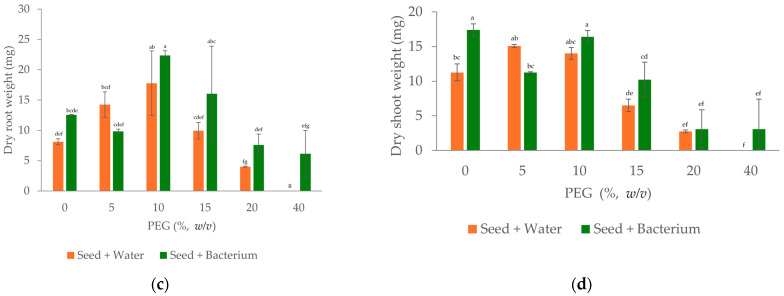
Effect of seed treatment with water or *Bacillus paralicheniformis* C1T-KM1901-B on the fresh and dry weight (mg) of roots and shoots in maize seedlings under different PEG concentrations. Fresh root weight (**a**), fresh shoot weight (**b**), dry root weight (**c**), and dry shoot weight (**d**). Bars represent mean ± SD (n = 3). Different letters above bars indicate significant differences according to one-way ANOVA followed by Fisher’s LSD test (*p* < 0.05).

Finally, the results in Figure 7 indicate that the response of maize seedlings obtained from maize seed treated with water or with *B. paralicheniformis* C1T-KM1901-B to PEG-induced drought stress depends on both the tissue analyzed (roots or shoots) and the PEG concentration. In the absence of PEG, seedlings obtained from seeds treated with *B. paralicheniformis* C1T-KM1901-B showed a significantly higher fresh weight (mg) of roots and shoots compared to the water-treated controls (Figure 7a,b; *p* < 0.05), suggesting a plant growth-promoting effect of the bacterium under conditions without simulated drought stress using PEG.

When evaluating the effect of PEG on seedlings grown from water-treated seeds (Figure 7a,b, orange bars), low PEG concentrations (5% and 10%) were associated with higher fresh weight (mg) of roots and shoots compared to the control without PEG, while higher PEG levels (15%, 20%, and 40%) caused a marked reduction in biomass. With 40% PEG, the water-treated seeds did not develop any detectable roots or shoots under the evaluated conditions. Changes in root and shoot dry weight (mg) followed a similar trend (Figure 7c,d).

In seedlings grown from bacteria-treated seeds (Figure 7a,b, green bars), the PEG concentration exerted a nonlinear effect on fresh biomass. While a reduction in fresh weight (mg) was observed with 5% PEG, the maximum values were recorded with 10% PEG, followed by a progressive decrease with higher concentrations. Statistical analysis (ANOVA followed by Fisher’s LSD, *p* < 0.05) indicates that the beneficial effect of *B. paralicheniformis* is evident under moderate drought stress (15% PEG), where inoculated seedlings showed significantly higher fresh root and shoot weights than uninoculated controls. Under severe drought stress (20–40% PEG), differences in fresh and dry biomass between treatments were generally not statistically significant; however, at 40% PEG, only seedlings derived from seeds maize treated with bacteria showed germination (Table 4), and limited root development. Regarding dry biomass (Figure 7c,d), statistically significant differences were detected mainly for shoot dry weight (mg) at 15% PEG, suggesting that bacterial inoculation primarily influences biomass maintenance under moderate drought stress.

## 3. Discussion

### 3.1. Distribution of Endophytic Microorganisms in Avicennia germinans

From a classical point of view, the presence of endophytes in plants is considered the resilient factor that allows these higher organisms to tolerate adverse environmental conditions [53,54]. In this context, although host plants may harbor a wide variety of endophytes, only those that provide specific benefits are essential for strengthening their resistance to these environmental challenges. Thus, the presence, type, pattern, distribution, colonization, and diversity of endophytes in host plants are all influenced by geographic location and environmental factors that result in the endophytic microbiome [9,55]. It was observed that the number of endophytic microorganisms isolated from *A*. *germinans* changed according to the sampling site (Figure 1). The greater environmental and saline stress in the mangrove ecosystem at site KM1901 may influence the reduction in plant height as supported by other studies [56,57,58], and in the number of isolated microorganisms compared to the other sampling sites (Table 1 and Figure 1). Additionally, this study showed differences in the tolerances to sodium chloride shown by the microorganisms isolated from the plant tissue from which they originate (Figure 2). It is known that endophytes in each organ of the plant are affected by the physiology and ecology of the plants [59]. Therefore, each organ offers endophytes a particular microenvironment, a selection factor for the communities established in each plant tissue. Similar results were found in *Salicornia europaea*, a halophile plant like *A*. *germinans*, in which beta-diversity analysis showed that endophytic bacterial communities were grouped according to natural and anthropogenic salinity sites and the plant organ, roots or shoots, suggesting that both local environmental factors and specific tissues significantly influence the structure of these communities [60].

### 3.2. Plant Growth—Promoting Traits of Halotolerant Endophytes

The role of endophytes in plant growth promotion includes producing phytohormones, nitrogen fixation, nutrient solubilization, or action against pathogens [56]. In this regard, in Table 2, the screening of several direct mechanisms tested with the most halotolerant endophytes obtained from *A*. *germinans* indicate that all isolates can produce phytohormones such as IAA in a range between 4.0 and 19.3 µg mL^−1^. These results are consistent with another study in which isolates identified as *Bacillus* sp., obtained from various halophilic plants (*Distichlis spicata*, *Cynodon dactylon*, *Eragrostis obtusiflora*, *Suaeda torreyana, Kochia scoparia*, and *Baccharis salicifolia*), reached an IAA production concentration of around 20 µg mL^−1^ with 15% NaCl [57]. Similar IAA production concentration (20 ± 0.7 µg mL^−1^) was reported for *B*. *licheniformis* isolated from *Vigna radiata* with an adaptive response to 15% NaCl [58]. These results show that halotolerant endophytes of the Bacillaceae family can produce auxins such as IAA. This IAA production could lead to direct benefits for the plant, such as regulating its growth and improving the ability of plants to access water and nutrients in saline soils.

Other aspects related to nutrient uptake are also shown in Table 2, where it is evident that all the isolates show proteolytic activity and most of them can fix nitrogen. High proteolytic activity has been reported for *Bacillus* sp. and *Streptomyces* sp., the dominant genera in mangrove ecosystems [61,62]. In plants, under conditions of low nitrogen availability, these bacteria could provide this essential nutrient to the plant from amino acids [63]. Regarding the solubilization of K and P, all tested isolates gave negative results for both. Other authors have also found that under laboratory conditions, the analyzed endophytes did not exhibit these characteristics [58,63].

On the other hand, among the indirect mechanisms of plant growth promotion, Figure 3 shows the characterization of the antagonistic activity of the most halotolerant endophytes isolated from *A*. *germinans* against four species of phytopathogenic fungi. The two isolates with the greatest antagonistic activity were C5H-SL01 and C3H-SL01-B2, which according to the literature, could use mechanisms such as hydrolytic enzymes like chitinases or the production of molecules such as natural antibiotics [64]. However, most isolates show less than 50% radial inhibition, especially against *Botrytis cinerea*, and *Aspergillus* sp. This low antagonistic capacity may be because mangroves represent environments with high selective pressure due to their salinity, oxygen-poor soils, variations in water availability, and exposure to sudden temperature changes. In this context, endophytic microorganisms do not necessarily exhibit strong antagonism but may prioritize coexistence and cooperation with other plant organisms [65].

### 3.3. Tolerance of Endophytic Isolates to PEG-Induced Drought Stress

The final characterization of endophytic microorganisms of *A*. *germinans* in this study involved evaluating the tolerance of the most halotolerant isolates to PEG-simulated drought stress (Figure 4). Although tolerance decreased with increasing PEG, with all isolates sensitive (≤0.3) at concentrations of 40%, two strains, C1T-KM1901-B and C2H-PV01, showed complete tolerance at 20%. Similar behavior was observed in bacterial isolates from Mungbean (*Vigna radiata* L. Wilczek) nodules, which revealed that the growth of bacterial isolates was negatively affected by an increase in drought stress from 10 to 40% of PEG and only 25% of 98 isolates could tolerate a high level of drought stress, i.e., 40% of PEG. Furthermore, the study revealed that tolerance to PEG stress was affected by temperature [66]. This highlights that tolerance to PEG is not a universal trait and may depend on the strain and the environment of origin.

### 3.4. Multivariate Analysis and Selection of Bacillus paralicheniformis C1T-KM1901-B

Principal component analysis (PCA) revealed functional separations among the evaluated traits, indicating that halotolerance, antagonistic activity, and IAA production are not uniformly expressed across all isolates. Isolates with high halotolerance tended to exhibit reduced antagonistic activity, likely due to metabolic compensation, in which resources are preferentially allocated to osmoprotection rather than the synthesis of antifungal secondary metabolites [67,68]. As mentioned previously, the extreme environment in which *A*. *germinans* grows may already be limiting for phytopathogenic fungi, favoring other traits in some bacteria. Furthermore, high IAA production was associated with increased sensitivity to severe osmotic stress (20% PEG), suggesting that the energy cost of auxin biosynthesis and active metabolism limit bacterial resilience under extreme drought conditions [33,42]. In this context, IAA-mediated plant growth promotion may be more effective under moderate stress conditions, while extreme water limitation favors metabolically conservative strategies. The C1T-KM1901-B isolate exhibited a balanced phenotype, clearly differentiating itself from the other isolates. C1T-KM1901-B combines high tolerance to NaCl (15%) and PEG (20%) with the potential to promote plant growth, supporting its selection for genomic sequencing and evaluation as a microbial inoculum for maize cultivation in saline and drought-prone environments.

### 3.5. Genomic Basis of Drought Tolerance and Plant Growth Promotion in Bacillus paralicheniformis C1T-KM1901-B

The genomic identification of the halotolerant endophytic isolate C1T-KM1901-B indicated that it corresponds to the species *B. paralicheniformis*. Based on reports in the literature for the genus *Bacillus* sp., several protein sequences present in this isolate were classified as related to drought stress tolerance and plant growth-promoting activity (Table 3). Confirmation of the presence of these sequences with bioinformatic tools suggests that, from a molecular point of view, the drought stress tolerance of this bacterium could be mainly due to the presence of genes associated with the production of osmoprotectants and the regulation of oxidative stress.

In the genome of *B*. *paralicheniformis* C1T-KM1901-B, we identified the genes coding for proteins *gbsA/B*, *spo0A*, *EpsA*, *proH*, *sodA*, *kat*, *blue/tpx*, *speE*, *entC*, *asbA*, and *dhbA* (Table 3), which are associated with osmoprotection, antioxidant response, and iron homeostasis. [68,69,70].

The *gbsA* and *gbsB* genes detected in *B*. *paralicheniformis* C1T-KM1901-B, are involved in glycine-betaine synthesis, which have been described in strains of *Bacillus subtilis* under high osmolarity stress [71,72]. In this study, the ability of *B*. *paralicheniformis* C1T-KM1901-B to tolerate 20% PEG-induced osmotic stress might be related to the activation of the corresponding genes under such conditions. Nonetheless, additional studies are required to confirm whether glycine-betaine effectively accumulates under osmotic stress conditions. In the *B*. *paralicheniformis* C1T-KM1901-B genomic analysis was also observed in the codification of genes for proteins related with endospore and biofilm formation, such as *spo0A*, a central transcriptional regulator that controls the expression of over 100 genes, including those required for biofilm matrix gene expression and sporulation. *Spo0A* and *EpsA*, which regulate sporulation and exopolysaccharide (EPS) synthesis, have also been described in endospore- and biofilm-producing strains of *B*. *subtilis,* respectively [32,34].

Regarding oxidative stress regulation, *B*. *paralicheniformis* C1T-KM1901-B harbors key protein sequences encoding antioxidant enzymes that counteract reactive oxygen species (ROS), including *sodA* (superoxide dismutase), *cat* (catalase), *btuE* and *tpx* (peroxidases) (Table 3). These genes have been reported previously in microbial endophytes under oxidative stress conditions [37,38,41,73].

On the other hand, in *B*. *paralicheniformis* C1T-KM1901-B, the sequences *speE*, *entC*, *dhbA* and *asbA* were identified (Table 3). These four genes have been reported to be responsible for polyamine production and siderophore synthesis, key processes in iron homeostasis and the regulation of the Fenton reaction mediated by oxidative stress [69]. Altogether, the genomic features identified in *B*. *paralicheniformis* C1T-KM1901-B support its capacity to synthesize osmoprotective and antioxidant compounds, reinforcing the connection between its genomic traits and the beneficial effects on host plants, as observed in the evaluation of its effect on maize seeds (Table 4).

Regarding plant growth promoting activities in *B*. *paralicheniformis* C1T-KM1901-B, genes sequences such as *fni*, *idi*, and *miaA* were identified (Table 3). These proteins are involved in the biosynthesis of isoprenoids required to produce cytokinins and carotenoid compounds [70]. In addition, genes sequences associated with the synthesis of acetoin (*alsS* and *alsD*) and 2,3-butanediol (*bdhA*) were identified in the *B*. *paralicheniformis* C1T-KM1901-B genome. These compounds are described as promoters of plant growth in environments subject to abiotic stress in the genus *Bacillus* [68,74]. However, in this study, *B*. *paralicheniformis* C1T-KM1901-B did not show Abscisic Acid (ABA)-associated proteins sequences (*CPS-KS*, *GA20ox*, and *GA3ox*), and for Jasmonic Acid (JA)-associated proteins sequences (*cyp112* and *cyp117*) showed less than 35% identity (Table S1).

With regard to solubilizing and capturing key elements such as phosphorus and iron, although the *B*. *paralicheniformis* C1T-KM1901-B strain did not show the ability to solubilize phosphorus and potassium in the laboratory, the genes responsible for the synthesis of proteins related to the mobilization and transport of phosphates, such as *phoA* and *gntP*, were identified. In addition, the genes coding for proteins *entC*, *dhbC*, and *feuC*, which are associated with the production of siderophores and iron transport [49,75], as well as *asbA*, related to the production of siderophores such as petrobactin, suggests an alternative pathway for iron acquisition, which is key in mitigating oxidative stress during drought conditions. These genomic features reinforce the potential of this halotolerant endophyte as a bioinoculant to improve agricultural productivity under abiotic stress conditions as demonstrated by the use of seed germination assays under drought stress.

### 3.6. Implications of the Use of Bacillus paralicheniformis C1T-KM1901-B as a Bioinoculant Under Drought Stress Conditions

The halotolerant endophytic isolate *B*. *paralicheniformis* C1T-KM1901-B, obtained from *A*. *germinans*, stands out both for its genomic potential associated with the production of osmoprotectants, regulation of oxidative stress, and promotion of plant growth and for its functional capacity to improve variables related to germination in maize seeds subjected to simulated drought stress with PEG. The results obtained show that the halotolerant endophyte *B*. *paralicheniformis* C1T-KM1901-B can exert a plant growth-promoting effect in the absence of PEG, as evidenced by an increase in stem dry biomass and greater water retention in both roots and stems of bioinoculated maize seedlings. Under extreme simulated water-stress conditions (40% PEG), the treatment of maize seeds with the bacterium significantly improved the germination potential (GP) and germination index of drought-resistant seeds (SGI-DR) (Table 4), suggesting a protective role for the endophyte during the early stages of germination under severe water limitations. In contrast, under mild drought stress conditions (5% PEG), a decrease in the fresh weight of roots and stems was observed compared to seedlings obtained from maize seeds treated with water alone. This behavior suggests a transient physiological response associated with an early osmotic adjustment, rather than a negative effect of the bioinoculant, since the fresh weight of the roots and stems is no different from seedlings obtained from untreated maize seeds (Figure 7a,b). However, this trend changes with increasing PEG concentration, indicating that the seed response to the interaction between the bioinoculant and drought stress is not linear [76]. Under low or extremely high drought stress conditions, seedlings can modulate the beneficial effect of the endophyte, either because bacterial adaptive mechanisms are not fully activated at low PEG concentrations or because the extreme stress partially exceeds the mitigation capacity of the plant-microorganism system [77,78]. Future studies should consider a larger number of seeds and biological replicates to confirm this trend and more robustly evaluate the nonlinear dynamics of the physiological response under different intensities of drought stress. The association with halotolerant endophytes can enhance plant resilience to abiotic stress through multiple complementary mechanisms, such as phytohormone production, osmolyte synthesis, and activation of antioxidant defense systems. Among these mechanisms, microbial production of indole-3-acetic acid (IAA) plays a particularly important role during seed germination and early seedling establishment. In this study, *B. paralicheniformis* C1T-KM1901-B produced 9.3 ± 4.1 µg mL^−1^ of IAA under in vitro conditions (Table 2), a concentration within the range reported to exert physiological effects on root development [15]. Bacterial IAA can stimulate cell elongation and division in emerging root tissues, promoting root protrusion, early root elongation, and lateral root formation [68]. These responses increase the effective root surface area, thereby improving water uptake and nutrient acquisition during the critical early stages of seedling establishment under saline or water-scarce conditions [73]. In addition to its role in modulating plant physiology, microbial IAA may also contribute to bacterial stress tolerance. Previous studies have shown that IAA can improve bacterial survival under osmotic stress, with significantly lower cell mortality in IAA-treated bacterial populations compared to untreated cells [42]. This dual function suggests that IAA acts as a signaling molecule in plant-microbe interactions and as a protective compound that enhances bacterial adaptation to adverse environmental conditions. All these physiological modulations could translate into significant increases in biomass, photosynthetic rate, and stomatal conductance, improving the plant’s adaptive capacity [37,79]. Therefore, the bacterium could optimize water absorption efficiency in maize seeds (Figure 7) and play a key role in reducing the adverse effects of osmotic stress, possibly through the regulation of metabolic pathways associated with the stress response [80]. We believe that this observation highlights the importance of integrating endophytes adapted to extreme environments into sustainable strategies that improve the resilience of non-halophytic crops, such as maize, to adverse abiotic conditions. Considering the growing challenges that climate change brings, leveraging endophytes such as *B. paralicheniformis* C1T-KM1901-B presents a practical solution for maintaining agricultural output in areas vulnerable to drought and soil salinity [81]. These findings highlight the importance of isolating halotolerant endophytes as a biotechnological tool to address agricultural challenges in climate change.

## 4. Materials and Methods

### 4.1. Sampling Sites and Sample Collection

**Figure 8:**
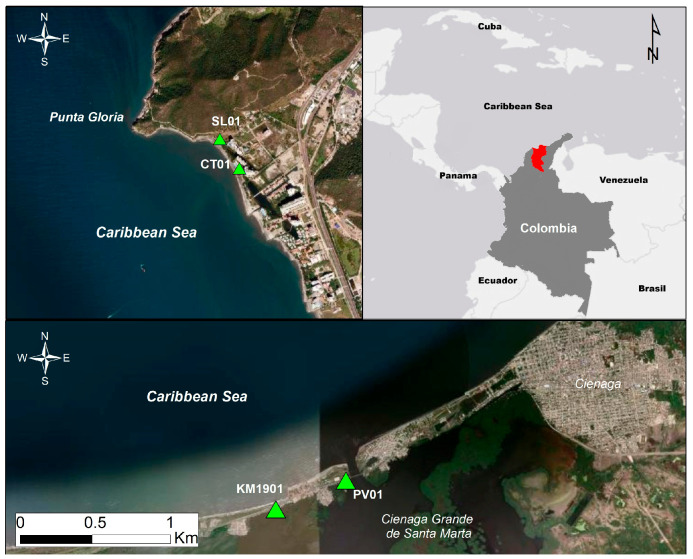
Geographic location of the four *A. germinans* sampling sites in the Colombian Caribbean. SL01 and CT01 are in the Pozos Colorados sector of Santa Marta, while PV01 and KM1901 are situated along the Ciénaga Grande de Santa Marta lagoon system. Green triangles mark the precise sampling points of *A. germinans* tissues. The red highlighted area represents the Department of Magdalena (Colombian Caribbean), where the sampling sites were selected.

The sampled mangrove ecosystems were located near the departments of Atlántico and Magdalena on the Colombian Caribbean coast, known as Sierra Laguna (SL01), Cabo Tortuga (CT01), Pueblo Viejo (PV01), and Km 19 (Km1901), and were identified with Google Earth Pro by the presence of *A*. *germinans* and salt stress (Figure 8). The four areas studied present related environmental dynamics: Sierra Laguna (SL01) and Cabo Tortuga (CT01) are in the Pozos Colorados, a touristic sector of Santa Marta city, characterized by sandy beaches bordered by waterfront residential condominiums, where urbanization has exerted pressure on adjacent mangrove ecosystems, generating surface loss and altered water flows. Further west, Pueblo Viejo (PV01) is located on the deltaic plain of the Magdalena River, with direct contact with the Cienega Grande of Santa Marta, an estuarine complex of high biological productivity where mangrove ecosystems have a riverine influence. Finally, the sector known as Km 19 (Km1901) is located on the road and coastal strip that connects Santa Marta with Cienega, in a section characterized by narrow beaches, coastal wetlands and equally close to the of Cienega Grande Santa Marta and where phenomena such erosion, sedimentation and salinity make this area vulnerable to human intervention and climate change [82,83].

Adult plants with no evidence of damage or disease were chosen at each location to decrease the likelihood of isolating pathogens. Three to five plants were chosen in drought and hyper-salinity conditions, and their GPS coordinates were collected (Table 1). In addition, a multiparameter probe (YSI EXO-1) was used to assess groundwater parameters such as temperature, salinity, dissolved oxygen, and pH at four sites throughout the study region. Samples of stems, leaves, flowers, propagules, pneumatophores, and internal roots (10–20 cm depth) were collected from each plant using sterilized equipment. The samples were packed in labeled sterile bags and maintained in a portable refrigerator at 4 °C to guarantee appropriate refrigeration [33]. They were promptly transported to the laboratory for processing.

### 4.2. Isolation of Halotolerant Endophytes from A. germinans

Sampled tissues were surface sterilized by extensive washing in tap water, followed by 80% ethanol (2 min), 4% sodium hypochlorite (5 min), and eight washed with sterile water. To confirm sterilization, aliquots of the last washing water were cultured on yeast extract-glucose agar (YGA) at 25°C for 72 h. Then, with a sterile scalpel, the bark of stems, propagules, and pneumatophores were removed, and the internal tissues were macerated in a sterile 0.9% saline solution. Leaves were macerated under the same conditions. The macerated liquid was cultured on YGA with 3.5% NaCl agar and incubated at 25 °C for 72 h [34]. Purified isolates were grown on Luria–Bertani (LB)-broth at 25 °C, and bacteria were stored in 30% (*v*/*v*) glycerol at −80 °C [84]. Isolates were named using a coding scheme combining letters and numbers to indicate their origin. “C” followed by a number represents the colony, while “R,” “T,” “H,” “P,” and “N” denote root, stem, leaf, propagule, and pneumatophore, respectively. Sampling sites are identified as “SL01” (Sierra Laguna), “CT01” (Cabo Tortuga), “PV01” (Pueblo Viejo), or “KM1901” (Kilometer 19). For instance, C2H-SL01 refers to the second colony from a leaf sampled at Sierra Laguna.

### 4.3. NaCl Tolerance

A colony of each isolated strain was exposed to various salinities (5%, 7.5%, 9.0%, 10%, 12.5%, and 15% NaCl) in LB broth cultures [85]. The cultures were incubated at 200 rpm/25 °C/5 days, observing turbidity and daily growth. Bacteria with the highest tolerance were chosen for further studies.

### 4.4. Characterization of Plant Growth-Promoting Activities by Endophytes

#### 4.4.1. Production of Indole Acetic Acid (IAA)

IAA production by the isolated bacterial strains was measured using Salkowski’s reagent, which contained 12 gL^−1^ of FeCl_3_ in 7.9 M H_2_SO_4_ [86]. The strains were cultured in the King B medium and incubated at 180 rpm/25 °C/48 h. After 48 h, the medium was centrifuged at 6000 rpm for 5 min, and 1 mL of the supernatant was mixed with 1 mL of the reagent and incubated for 30 min at room temperature in the dark. Finally, the absorbance was measured at 530 nm in a UV/Vis spectrophotometer-single beam Mapada—Model P1. A standard curve was constructed using 98% IAA (Sigma-Aldrich, St. Louis, MO, USA) to determine the concentration of IAA in the samples from their absorbance. For this purpose, known concentrations of IAA of 0–150 μg mL^−1^ were taken and mixed with Salkowski’s reagent, and its absorbance at 530 nm was measured [86]. The assays were performed in triplicate.

#### 4.4.2. Solubilization of Phosphates and Potassium, Proteolytic Activity and Growth in Nitrogen-Poor Medium

Phosphate solubilization, potassium solubilization, proteolytic activity, and growth in nitrogen-limited medium, were qualitatively assessed by observing halo formation or microbial growth on specific culture media. Phosphate solubilization was evaluated on Pikovskaya’s agar with the following composition (gL^−1^): yeast extract 0.5, glucose 10, Ca_3_(PO_4_)_2_ 5.0, (NH_4_)_2_SO_4_ 0.2, KCl 0.1, MgSO_4_ 0.001, MnSO_4_ 0.0001, FeSO_4_ 0.0001, and agar 18 [85]. Potassium solubilization capacity was tested on Aleksandrov’s medium with the following composition (gL^−1^): glucose 5, MgSO_4_·7H_2_O 0.5, FeCl_3_ 0.005, CaCO_3_ 0.1, CaHPO_4_ 2, KAlSi_3_O_8_ 2, and agar 18 [87]. Proteolytic activity was determined using a medium with the following composition (gL^−1^): tryptone 5, yeast extract 2.5, glucose 1, NaCl 2.5, agar 18, and pH 7.0. Additionally, 100 mL of skimmed milk was added to the medium after sterilization [88]. Growth in the nitrogen-poor medium was assessed using a semisolid medium containing (gL^−1^): mannitol 5.0, K_2_HPO_4_ 0.6, KH_2_PO_4_ 1.8, MgSO_4_·7H_2_O 0.2, NaCl 0.1, CaCl_2_·2H_2_O 0.02, yeast extract 0.05, and agar 1.8 [89,90]. All cultures were incubated for 72 h at 25 °C, and assays were performed in triplicate.

#### 4.4.3. Antagonism Tests Against Phytopathogenic Fungi

The phytopathogenic fungi *Alternaria* sp., *Fusarium* sp., *Aspergillus* sp., and *Botrytis cinerea* were donated by the Colombian Corporation for Agricultural Research, AGROSAVIA, and the Research Group: Biological Chemistry: Biosynthetic Design of Fungicides at the University of Cadiz. These were reactivated by culturing them on PDA and incubating them at 25 °C for 7 days. Subsequently, the antagonism of the isolated bacteria against these fungi was evaluated by inoculating them in a PDA medium, inoculating four streaks 1 cm from the edge of the Petri dishes. Then, a 5 mm disk of mycelium of the fungi, with 5 days of growth, was placed in the center of the plates, which were incubated at 25 °C for 6 days. The antagonistic effect was calculated using the equation:

(1) $$Radial\;inhibition\;\left(\%\right)=\left(\frac{R_{c}-R_{i}}{R_{c}}\right)\times 100$$

*R_c_* is the fungus’s radius without the bacteria, and *R_i_* is the radius in the presence of the antagonistic bacteria [91]. All experiments were performed in triplicate.

### 4.5. Screening of Halotolerant Endophytic Bacteria Strains for Drought Tolerance

LB broth was prepared with different concentrations (0%, 5%, 10%, 20%, and 40%) of polyethylene glycol 6000 (Sigma-Aldrich, St. Louis, MO, USA) to obtain different levels of water potential [92]. The culture media were inoculated at each concentration with mid-logarithmic phase bacterial cells fixed at OD_600_ equal to 0.2. The inoculated cultures were incubated at 150 rpm 28 °C 72 h. Bacterial growth was estimated by measuring the optical density at 600 nm with a UV/Vis spectrophotometer-single beam Mapada—Model P1 at 0, 24, 48, and 72 h. Data was obtained from three independent cultures. Drought tolerance was classified based on OD values as follows: sensitive (≤0.3), tolerant (0.4–0.6), and completely tolerant (>0.6) [93].

### 4.6. Genomic DNA Extraction and Sequencing of Strain C1T-KM1901-B

Genomic DNA was extracted by culturing the bacterial strain C1T-KM1901-B in tryptone soy broth (TSB), followed by cell lysis with SDS and proteinase K. Purifications were performed using organic phases (phenol: chloroform and CTAB/NaCl) and precipitation with isopropanol and ethanol, obtaining pure DNA resuspended in MilliQ water (Milli-Q system, Merck Millipore, Darmstadt, Germany) [94].

The DNA concentration was determined for genomic sequencing with a Qubit v.2.0 fluorometer (Invitrogen, Waltham, MA, USA). MicrobesNG (University of Birmingham, Birmingham, UK) performed library preparation and DNA sequencing. Libraries were performed using the Nextera XT kit (Illumina, San Diego, CA, USA) following the manufacturer’s protocol.

Sequencing was performed using an Illumina MiSeq. Reads were trimmed using Trimmomatic v.0.36 [95], and quality tested using FastQC v.0.11.8. Genome assembly was performed using SPAdes v.3.12.0 [96], and the resulting contigs were extended and joined into scaffolds using SSPACE v.2.1.1 [97]. The gaps generated were closed using GapFiller v.1-10 [98]. The genome assembly quality was assessed using Quast v.5.2 [99], and genome purity was verified using the Microbial Genome Atlas web service v.1.3.9.0 [100]. Taxonomic classification is processed using average nucleotide identity (ANI) and average amino acid identity (AAI), calculated with both the best one-way and two-way hits between genomic and protein sets [101,102].

The complete genome was annotated using Prokka v1.12 [103]. A detailed search was performed for genes associated with tolerance to abiotic stress and plant growth-promoting activity (Supplementary Table S2). To this end, a database with protein sequences related to these genes obtained from UniProt database was compiled. These sequences were compared with the amino acid sequences annotated in the genome using Blastp (NCBI BLAST+), considering as positive those with identity greater than 35%, query coverage greater than 50%, and an E-value less than 10^−5^.

### 4.7. Evaluation of Drought Stress Tolerance in Maize Seeds Germination Using PEG and A. germinans Endophytes

#### 4.7.1. Selecting and Treatment of Maize Seeds

Full-grained Maize seeds of the same size, without holes caused by worms or insects or any sign of alteration or contamination, were selected. A 70% ethanol solution was used to disinfect the seeds for 2 min and then rinsed with sterile distilled water thrice. The disinfected seeds were placed for 24 h in sterile distilled water at a rate of 100 seeds/100 mL for total imbibition at 25 °C and 300 rpm. Another set of 100 disinfected seeds was placed for 24 h for total imbibition in a bacterial suspension with the endophytic strain to be evaluated at a concentration of 1 × 10^6^ CFU mL^−1^ at a rate of 100 seeds: 100 mL under shaking conditions at 300 rpm and 25 °C [104].

#### 4.7.2. Maize Seed Germination Under Drought Stress Conditions by PEG

PEG 6000 was used as a stress agent to simulate in vitro drought stress conditions of Maize seeds [76]. For this purpose, PEG 6000 solutions were prepared with distilled water at different concentrations of 0, 5, 10, 15, 20, and 40% (*w*/*v*). Each treatment was conducted in a germination dish containing 20 seeds soaked in water or bacterial suspension. The seeds were arranged in an organized manner on filter paper. The filter paper was moistened with 10 mL treatment solution, ensuring the seeds were moistened with the solutions. The seeds were kept for one night and one day. Each day, the treatment solution with a new PEG solution of the same volume and amount of concentration or water volume water was replaced in each germination container [105]. The seeds were kept under natural photoperiod conditions at 25 °C. The germination test was terminated when no seeds had germinated for 3 days. The germination monitoring period was 5 days [106].

#### 4.7.3. Measurement of Maize Seed Germination Variables

The germination rate of maize seeds was measured daily for five days, calculating the percentage of germinated seeds in total. Germination potential was calculated as the percentage of germinated seeds at the germination peak. After five days, shoot and root lengths were measured with a ruler, considering the total stem length as the sum of hypocotyl and epicotyl. Selected seedlings were measured the fresh and dry weight (mg) of shoots and roots; the dry weight (mg) was obtained after drying at 105 °C for 2 h, followed by 3 days at 80 °C. The root-shoot ratio was calculated from these measurements. The drought resistance coefficient was estimated by dividing the index of water- stress by the index of normal conditions. Finally, the germination readiness and vigor indices under drought were calculated using formulas that relate the number of seeds germinated on different days and the average shoot length on the fifth day [105].

### 4.8. Statistical Analysis

Experiments were performed in a fully randomized design in triplicates. Data were tested for normality by the Shapiro–Wilk test (*p* ≥ 0.05) and for variance homogeneity by Levene’s test (*p* ≥ 0.05). One-way analysis of variance (ANOVA) followed by Fisher’s Least Significant Difference (LSD) test (*p* < 0.05) was applied to evaluate differences among treatments for germination percentage, germination potential (GP), seed germination index under drought resistance (SGI-DR), seed vigor index under drought resistance (SVI-DR), root–shoot ratio (Table 4), as well as for fresh and dry root and shoot biomass under different PEG concentrations (Figure 7). Analyses were performed using the software Minitab^®^ Statistics v. 18. Data are expressed as mean ± standard deviation (SD). Statistical differences among treatments were determined by one-way ANOVA followed by Fisher’s LSD test at *p* < 0.05.

## 5. Conclusions

In this study, *Avicennia germinans*, a plant from coastal mangrove ecosystems of the Colombian Caribbean, was used as a source of halotolerant endophytic bacteria. Sixty-eight bacteria were isolated, all Gram-positive and endospore-forming. These salt-tolerant endophytic bacteria (minimum 5% sodium chloride) also showed tolerance to simulated drought with up to 20% PEG and exhibited plant growth-promoting characteristics, such as indoleacetic acid (IAA) production. Among them, the isolate C1T-KM1901-B, which showed the highest tolerance to both sodium chloride (15%) and PEG-induced drought stress (20%), significantly increased the germination percentage of maize seeds, even under extreme drought conditions (40% PEG). In addition, seedlings derived from seeds inoculated with this isolate exhibited significantly higher fresh root and shoot weight, as well as higher dry root weight compared to non-inoculated controls under moderate drought stress (15% PEG). The strain C1T-KM1901-B was genetically identified as *Bacillus paralicheniformis*. Its genome revealed a genetic profile with the potential to produce osmoprotective substances, regulate oxidative stress, and promote plant growth. These results demonstrate the potential of *A*. *germinans* as a promising source of bioinoculum to improve the yield of crops, such as maize, that are susceptible to saline and drought stress in diverse environments.

## Figures and Tables

**Table 1:** Physicochemical Characteristics of Sampling Sites, Plant, Water, and Soil Parameters.

Sampling Site Code	Sampling Site Name	Georeference	Sampled Organs ^1^	Water Table Level (cm)	EC-GW ^2^ (mS cm^−1^)	Plant Height (m)	Soil Type	Soil pH	ECs ^3^ (mS cm^−1^)	SWC ^4^ (%)
SL01	Sierra Laguna	11°10′25″ N, 74°12′59″ W	L, S, R, P, and Se	68.00	106.30	3.20	Clayey	8.40	17.15	65.00
CT01	Cabo Tortuga	11°10′30″ N, 74°14′09″ W	L, S, R, F, P, and Pr	57.00	85.74	2.50	Sandy	8.40	17.64	10.00
KM1901	Km 19, B/quilla–Sta. Marta	11°00′44″ N, 74°36′46″ W	L, S, R, and P	20.00	146.40	2.50	Sandy	7.86	41.50	46.60
PV01	Pueblo Viejo	10°59′18″ N 74°17′31″ W	L, S, R, F, and P	68.00	85.02	3.30	Sandy	8.40	8.56	40.40

^1^ L, leaves; S, stems; R, roots; F, flowers; P, pneumatophores; Pr, propagules; Se, Seedling. ^2^ EC-GW, Electrical Conductivity of Groundwater—a measure of salinity level in groundwater samples. ^3^ ECs, Electrical Conductivity of Soil—a measure of soil salinity based on soluble ions. ^4^ SWC, Soil Water Content—the percentage of water retained in soil samples.

**Table 2:** Plant Growth-Promoting Activity of Halotolerant Endophytes at 12% and 15% Sodium Chloride.

Isolate ID	Plant Tissue	NaCl Halotolerance	IAA ^1^ (µg mL^−1^)	Proteolytic Activity	Phosphate Solubilization	Potassium Solubilization	GNM ^2^
C2R-SL01_A	Root	12%	13.0 ± 1.0	+	−	−	+
C2R-SL01_B	Root	12%	4.0 ± 1.4	+	−	−	−
C4R-SL01_B	Root	12%	11.7 ± 3.3	+	−	−	+
C3R-CT01-C	Root	12%	11.7 ± 0.9	+	−	−	+
C1N-SL01	Pneum.	12%	5.3 ± 4.4	+	−	−	+
C1T-CT01	Stem	15%	16.3 ± 3.9	+	−	−	+
C2T-CT01	Stem	15%	15.7 ± 0.5	+	−	−	+
C1T-KM1901-B	Stem	15%	9.3 ± 4.1	+	−	−	−
C1H-SL01	Leave	12%	14.0 ± 1.4	+	−	−	−
C3H-SL01-B	Leave	12%	14.7 ± 1.9	+	−	−	+
C5H-SL01	Leave	12%	15.0 ± 1.4	+	−	−	+
C1H-PV01	Leave	12%	14.7 ± 2.5	+	−	−	+
C2H-PV01	Leave	12%	9.0 ± 3.6	+	−	−	−
C3H-PV01	Leave	12%	10.0 ± 4.5	+	−	−	−
C1FCT01	Flower	15%	11.0 ± 2.2	+	−	−	+
C1P-CT01-A	Propag.	12%	12.0 ± 5.7	+	−	−	+
C2P-CT01-B	Propag.	12%	17.3 ± 6.5	+	−	−	+
C1PL-SL01	Seedling	12%	19.3 ± 1.7	+	−	−	+

^1^ The concentration of IAA (µg mL^−1^) is given as the average value followed by its standard deviation (e.g., 13.0 ± 1.0). The symbols ‘+’ and ‘−’ in other columns represent positive or negative test or growth, respectively. ^2^ GNM, Growth in Nitrogen-limited Medium.

**Table 3:** Protein sequences associated with drought stress tolerance and plant growth-promoting activity from literature review identified in the genome of *Bacillus paralicheniformis* C1T-KM1901-B.

Protein Product	Protein Code	Gene	Refs.
Production of Osmoprotectants and Osmotic Regulation			
Glycine betaine transporter	SNY76213.1	*opuB*	[29]
Glycine betaine-binding lipoprotein	AGG59643.1	*opuAC*	[29]
Trehalose-6-phosphate hydrolase	SPY10133.1	*otsB*	[29]
Glucose-specific phosphotransferase component	ARC73959.1	*crr*	[30]
Phosphoglucomutase	SMF21391.1	*pgcA*	[30]
UTP–glucose-1-phosphate uridylyltransferase	SPY15128.1	*gtaB*	[30]
UDP-glucose 4-epimerase	CON87715.1	*galE*	[30]
Glucose-6-phosphate isomerase	WP_226567401.1	*pgi*	[30]
Glutamine–fructose-6-phosphate aminotransferase	CAK2235500.1	*glmS*	[30]
Phosphoglucosamine mutase	SPY19784.1	*glmM*	[30]
Bifunctional protein GlmU	NP_387931.1	*glmU*	[30]
UDP-N-acetyl-D-glucosamine dehydrogenase	QHM09130.1	*wbpA*	[30]
UDP-N-acetylglucosamine 2-epimerase	NP_391446.1	*mnaA*	[30]
UDP-glucose 6-dehydrogenase TuaD	AOL99425.1	*tuaD*	[30]
Gamma-glutamyl phosphate reductase	KOS72353.1	*proA*	[31]
Pyrroline-5-carboxylate reductase	AOR98725.1	*proH*	[31]
Protein-tyrosine kinase modulator EpsA	NP_391317.1	*epsA*	[32,33,34]
Protein-tyrosine kinase modulator EpsB	NP_391316.1	*epsB*	[32,33,34]
Stage 0 sporulation protein A	CAB14353.1	*spo0A*	[32,33,34]
Aspartate kinase	WP_335451403.1	*lysC*	[31,35]
Aspartate-semialdehyde dehydrogenase	SNY63598.1	*Asd*	[31,35]
Diaminobutyrate–2-oxoglutarate transaminase	WGP06652.1	*ectB/dat*	[31,35]
Glycine betaine aldehyde dehydrogenase	XLG11555.1	*gbsA*	[31,35]
Choline dehydrogenase	SNY62559.1	*gbsB*	[31,35]
Betaine aldehyde dehydrogenase	RUS10360.1	*codA*	[31,35]
Glucose-1-phosphate adenylyltransferase	AOR99382.1	*glgC*	[31,35]
1,4-alpha-glucan branching enzyme	AOR99383.1	*glgB*	[31,35]
ROS Regulation and Regulation of Genes Detoxifying ROS			
Transcriptional regulator sensing organic peroxides	NP_389198.1	*ohrR*	[29]
Nitrate reductase	KOS73226.1	*narG*	[36]
Catalase	SPY10020.1	*katE*	[36,37]
Peroxidase	AKD35422.1	*btuE*	[37]
Thiol peroxidase	KJJ43384.1	*Tpx*	[38]
Monooxygenase (cytochrome P450)	KIU12180.1	*Cyp*	[38]
Cytochrome P450 oxidoreductase	AGA20975.1	*CYP120A1*	[38]
Spermidine synthase	SPY11098.1	*speE*	[38]
Superoxide dismutase	SPY11823.1	*sodA*	[39]
Alkyl hydroperoxide reductase	KOS72958.1	*ahpC*	[39]
S-methyl-5-thioribose-1-phosphate isomerase	AOR97830.1	*mtnA*	[40]
Plant Growth Promoting Activity			
Adenine phosphoribosyltransferase	WOY75590.1	*apt*	[37,38,41]
tRNA dimethylallyltransferase	SNY63384.1	*miaA*	[42]
Isopentenyl-diphosphate Delta-isomerase	AOR98607.1	*fni/idi/ypgA*	[42]
Isopentenyl pyrophosphate isomerase	SPY12006.1	*fni*	[42]
3-hydroxybutyryl-CoA dehydrogenase	CON28795.1	*hbd*	[43,44]
gamma-DL-glutamyl hydrolase	WP_336804525.1	*pgdS*	[45]
Glutamate racemase	AEB64457.1	*racE*	[45]
alpha-acetolactate synthase	NP_391482.2	*alsS*	[46]
alpha-acetolactate decarboxylase	WP_243499356.1	*alsD*	[46]
L-threonine 3-dehydrogenase	NP_388505.1	*bdhA*	[46]
Acquisition of Essential Minerals (K, P and Fe)			
phosphatase	KOS73323.1	*phoA*	[47]
gluconate permease	BAA06503.1	*gntP*	[48]
sodium/malate symporter MaeN	QJP89869.1	*maeN*	[48]
Putative malate transporter YflS	BAA22312.1	*yflS*	[48]
dhbC isochorismate synthase (siderophore)	CAL0280723.1	*dhbC*	[49]
Isochorismatase	SPY15243.1	*dhbB*	[49]
2,3-dihydro-2,3-dihydroxybenzoate dehydrogenase	QSG00654.1	*dhbA*	[49]
2,3-dihydroxybenzoate-AMP ligase	SPY15244.1	*dhbE*	[49]
iron-uptake protein	NP_388042.2	*feuC*	[49]
ferri-bacillibactin esterase BesA	WP_144453004.1	*besA*	[49]
isochorismate synthase EntC	AVL04089.1	*entC*	[50,51]
2,3-dihydro-2,3-dihydroxybenzoate dehydrogenase	AOR99492.1	*entA*	[50,51]
2,3-dihydroxybenzoate-AMP ligase	AOS69206.1	*entE*	[50,51]
petrobactin biosynthesis protein AsbA	WWO35711.1	*asbA*	[52]

**Table 4:** Effect of the Endophyte *B*. *paralicheniformis* C1T-KM1901-B from *A*. *germinans* and Different PEG Concentrations on Seed Germination and Drought Resistance.

Seed Treatment	PEG (%)	Germination (%)	GP (%)	SGI-DR	SVI-DR	Root-Shoot Ratio
Seed + water	0	50 ± 7 ^cde^	50 ± 7 ^ab^	1.0 ± 0.1 ^bc^	1.0 ± 0.2 ^ab^	0.50 ± 0.04 ^d^
Seed + *B. paralicheniformis*	0	72.5 ± 3.5 ^abc^	47.5 ± 3.5 ^ab^	1.2 ± 0.1 ^abc^	1.4 ± 0.3 ^a^	0.68 ± 0.11 ^bcd^
Seed + water	5	52.5 ± 2.4 ^bcde^	40 ± 0.5 ^bcd^	1.0 ± 0.2 ^bcd^	1.0 ± 0.2 ^ab^	0.76 ± 0.08 ^abcd^
Seed + *B. paralicheniformis*	5	80 ± 14 ^ab^	62.5 ± 17.7 ^a^	1.3 ± 0.4 ^ab^	0.87 ± 0.3 ^b^	0.73 ± 0.03 ^abcd^
Seed + water	10	65 ± 14 ^abcd^	52.5 ± 3.5 ^ab^	1.4 ± 0.1 ^abc^	1.0 ± 0.1 ^ab^	0.99 ± 0.17 ^abc^
Seed + *B. paralicheniformis*	10	85 ± 7 ^a^	62.5 ± 10.6 ^a^	1.5 ± 0.3 ^a^	1.3 ± 0.4 ^a^	10.5 ± 0.07 ^ab^
Seed + water	15	60 ± 14 ^abcd^	45 ± 14 ^abc^	1.0 ± 0.3 ^bcd^	0.41 ± 0.14 ^c^	1.2 ± 0.2 ^a^
Seed + *B. paralicheniformis*	15	77.5 ± 10.6 ^abc^	55 ± 7^ab^	1.27 ± 0.04 ^abc^	0.85 ± 0.14 ^b^	10.7 ± 0.1 ^ab^
Seed + water	20	55 ± 7.1 ^bcde^	40 ± 0^bcd^	0.88 ± 0.08 ^cde^	0.07 ± 0.02 ^cd^	1.3 ± 0.4 ^abc^
Seed + *B. paralicheniformis*	20	37.5 ± 17.7 ^de^	25 ± 7 ^d^	0.53 ± 0.05 ^e^	0.09 ± 0.04 ^cd^	1.0 ± 0.0 ^abc^
Seed + water	40	0 ± 0 ^f^	0 ± 0 ^e^	0 ± 0 ^f^	0 ± 0 ^d^	0 ± 0 ^e^
Seed + *B. paralicheniformis*	40	27.5 ± 10.6 ^ef^	27.5 ± 10.6 ^cd^	0.55 ± 0.21 ^de^	0.007 ± 0.004 ^d^	0.55 ± 0.636 ^cd^

The values presented in the table represent the mean ± standard deviation (SD) from triplicate experiments. Letters indicate the grouping of means based on Fisher’s LSD test. This means that sharing at least one letter is not significantly different, whereas sharing no letters is statistically different (*p*-value < 0.05). GP, germination potential; SGI-DR, seed germination index under drought resistance; SVI-DR, seed vigor index under drought resistance.

## Data Availability

The original contributions presented in this study are included in the article/Supplementary Material. Further inquiries can be directed to the corresponding authors.

## Supplementary Materials

The following supporting information can be downloaded at https://www.mdpi.com/article/10.3390/plants15010143/s1, Table S1: Genome characteristics and assembly quality of *Bacillus paralicheniformis* C1T-KM1901-B. Table S2: Analysis of the presence of protein sequences associated with drought stress tolerance and plant growth-promoting activity from literature review identified in the genome of *Bacillus paralicheniformis* C1T-KM1901-B.
