# Supplementary material for: Increased Drought Stress Tolerance in Maize Seeds by Bacillus paralicheniformis Halotolerant Endophytes Isolated from Avicennia germinans
Source: Plants (Basel). 2026 Jan 4;15(1):143. doi: 10.3390/plants15010143 (PMC12788130; doi:10.3390/plants15010143)
Supplement: Supplementary file 1 [file plants-15-00143-s001.zip › Table S1. Genome characteristics, assembly quality, and genomic relatedness of Bacillus paralicheniformis C1T-KM1901B.pdf]

Table S1. Genome characteristics, assembly quality, and genomic relatedness (ANI) of *Bacillus paralicheniformis* C1T-KM1901B

A. Genome characteristics*

Metric	Value
Organism	*Bacillus paralicheniformis* strain C1T-KM1901B
Genome size (bp)	4,279,900
GC content (%)	45.72
Total genes	4,420
Coding sequences (CDS)	4,341
rRNA genes	9
tRNA genes	69
tmRNA	1

B. Assembly quality statistics

Metric	Value
Number of contigs	27
Contigs ≥ 0 bp	49
Contigs ≥ 1000 bp	24
Largest contig (bp)	586,963
Total length (bp)	4,279,155
Total length (≥ 0 bp)	4,284,291
Total length (≥ 1000 bp)	4,277,052
N50 (bp)	351,296
N75 (bp)	296,556
L50	5
L75	8
Number of N's	0
N's per 100 kbp	0

C. Average Nucleotide Identity (ANI) between C1T-KM1901B and the closest reference genome

Metric	Value
OrthoANIu (%)	**98.83**
Genome A length (C1T-KM1901B, bp)	3,711,780
Genome B length (reference, bp)	3,629,160
Average aligned length (bp)	2,708,747
Genome A coverage (%)	72.98
Genome B coverage (%)	74.64

D. OrthoANIu comparison matrix between C1T-KM1901B and reference *Bacillus paralicheniformis* strains

Query genome	S127	ATCC 12759	BL-09	KJ-16 (T)	ATCC 9945a
**C1T-KM1901B**	**96.58**	**96.52**	**96.57**	**96.57**	**96.64**
B. paralicheniformis S127	—	99.00	99.08	99.07	99.50
B. paralicheniformis ATCC 12759	96.29	—	99.26	99.28	98.94
B. paralicheniformis BL-09	98.18	98.89	—	98.92	99.03
B. paralicheniformis KJ-16 (T)	96.25	98.72	98.75	—	99.34
B. paralicheniformis ATCC 9945a	96.43	98.84	99.03	99.34	—

*The genome was assembled using SPAdes in individual mode with paired-end reads. The draft genome shows high quality (N50 = 351,296 bp; no ambiguous bases). ANI values >95% confirm that C1T-KM1901B belongs to the species *Bacillus paralicheniformis*, while differences among matrices indicate it is a genetically distinct environmental isolate associated with mangrove ecosystems.
